# An unusual case of a giant fetal facial tumour and review of the literature

**DOI:** 10.1007/s11845-023-03344-3

**Published:** 2023-03-25

**Authors:** Alex O. Start, Gillian A. Ryan, Barbra Cathcart, Jennifer Geraghty, Niamh Adams, Gabrielle Colleran, Claudine Vavasseur, John Caird, Dylan Murray, Jennifer M. Walsh

**Affiliations:** 1 Fetal Medicine, National Maternity Hospital, Holles Street, Dublin 2, Ireland; 2 UCD Perinatal Research Centre, School of Medicine, University College Dublin, Dublin, Ireland; 3 Department of Neonatology, National Maternity Hospital, Dublin 2, Ireland; 4 Paediatric Radiology, National Maternity Hospital, Dublin 2, Ireland; 5 Neurosurgery, Children’s University Hospital, Dublin 1, Ireland; 6 Plastic Surgery, Children’s University Hospital, Dublin 1, Ireland

**Keywords:** Congenital tumour, Fetal anomaly, Fetus, Pregnancy

## Abstract

We present the case of a pregnant 32-year-old woman who presented with a giant fetal facial tumour at 22 weeks. The mass, initially 4 × 3.5 × 3 cm in size, was largely cystic with a small solid component. It subsequently increased to 9 × 9 × 10 cm. Significant compression effects on the fetal orbit, temple and infratemporal fossa, with potential compression of the optic nerve, were noted on ultrasound and MRI. The cyst required drainage twice in the pregnancy: firstly to reduce the compression effects and secondly to facilitate caesarean delivery. Postnatally, the baby had significant compression and displacement of the craniofacial skeleton from the mass effect. Postnatal histology revealed a diagnosis of a teratoma. This case highlights the complexities and challenges surrounding the diagnosis and management of a giant fetal facial tumour.

## Introduction and case presentation

We present the complex case of a giant fetal facial tumour diagnosed at 22 weeks. There is a paucity of published data on both the antenatal and postnatal management of these large lesions. This case highlights the challenges of managing such cases, from antenatal diagnosis to delivery and the postnatal surgical management of such tumours. This case proved the importance of significant multidisciplinary team (MDT) involvement to ensure a good clinical outcome. The MDT included paediatric radiology, paediatric otorhinolaryngology, fetal medicine, neonatology and paediatric neurosurgery. It also adds to the body of literature on these unusual cases. Written consent has been obtained for the publication of this report with images.

### Case

We present the case of a pregnant 32-year-old woman who presented in her second pregnancy. She had a history of one previous uncomplicated pregnancy with a term vaginal delivery and a birth weight of 2900 g. There was no relevant past medical history.

**Fig. 1:**
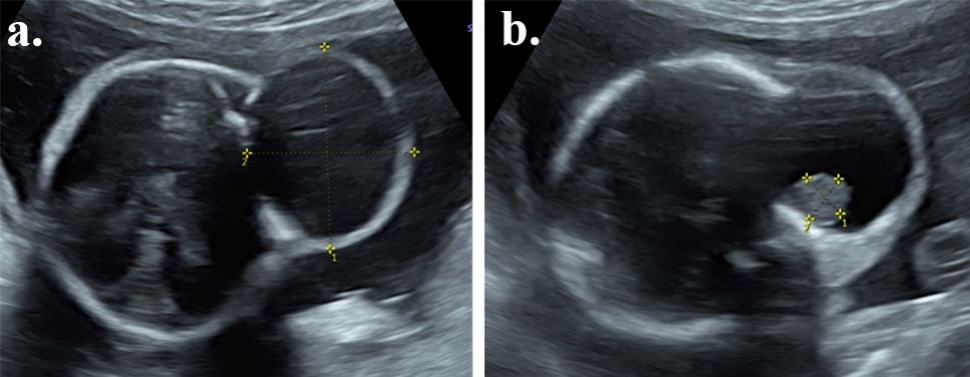
Ultrasound at 22 weeks’ gestation. **a** and **b** are ultrasound images of the lesion at 22 weeks gestation. The lesion (**a**) measures 4 × 3.5 × 3 cm in size with a single solid component of 1.02 × 1.03 cm size seen in (**b**)

**Fig. 2:**
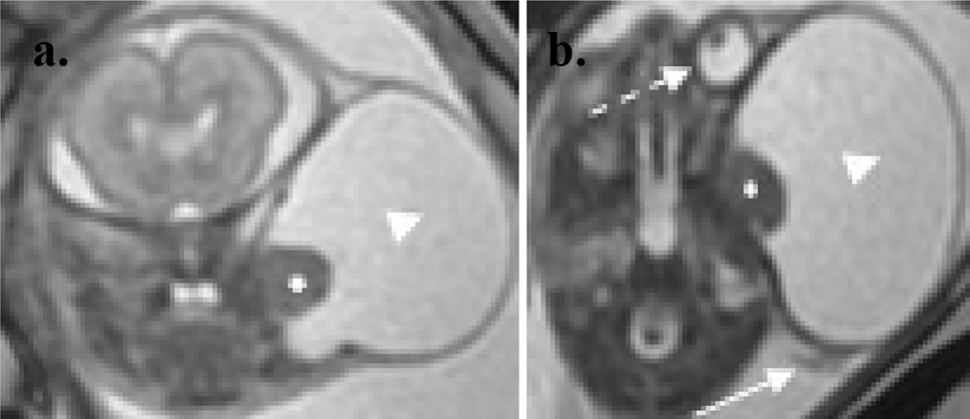
MRI at 23 weeks gestation. Coronal **a** and axial **b** T2-weighted MRI of the fetal head and neck demonstrate a large cystic lesion (arrowhead) in the left infratemporal region with a smaller solid component in its medial aspect. It is causing significant distortion of surrounding structures including displacement of the left orbit (dashed arrow) and ear (arrow)

A 22-week anatomy scan revealed a mass in the left temporal region measuring 4 × 3.5 × 3 cm (Fig. 1a). Further fetal medicine assessment confirmed the presence of a fetal facial lesion, extending from the left temporal region, with no significant flow on colour Doppler examination and with a single solid component measuring 1.02 × 1.03 cm (Fig. 1b), deep in the infratemporal fossa. A subsequent fetal MRI confirmed a large temporal mass (Fig. 2a, b). The differential diagnoses at the time included a lymphatic malformation, cystic teratoma, a branchial cyst/cleft remnant, congenital scalp haemangioma or a temporal bone meningocele/encephalocele. The possibility of arrested development of a conjoined twin was also considered.

**Fig. 3:**
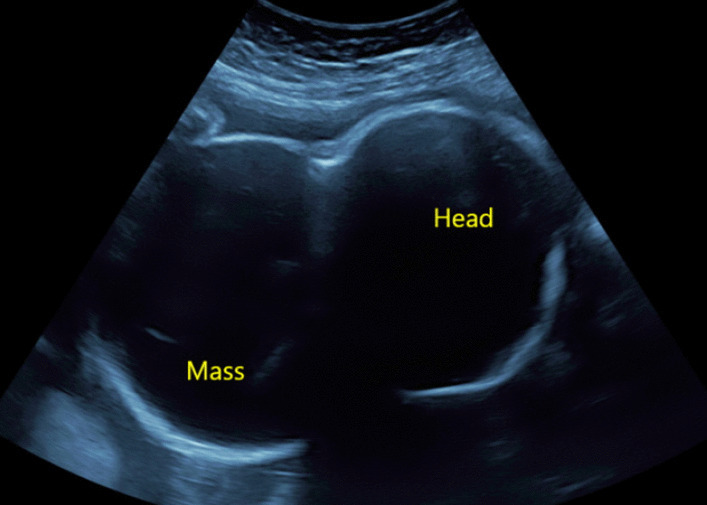
Ultrasound showing the enlarged lesion at 30 + 2 weeks gestation. This shows an ultrasound image of the mass at 30 + 2 weeks gestation. The tumour now measures 9 × 9 × 10 cm

**Fig. 4:**
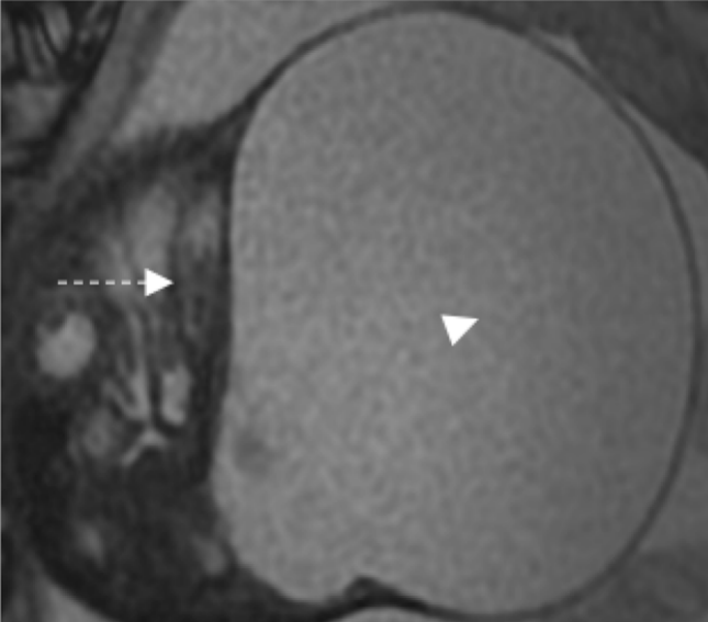
Fetal MRI at 31 weeks gestation. Axial T2-weighted fetal MRI demonstrates interval increase in the size of the large cystic mass (arrowhead) with increased mass effect on the left orbit (dotted arrow)

The patient was closely monitored with regular ultrasound scans. By 28 + 3 weeks, the cystic mass began to increase in size to 8.5 × 7.7 × 7.5 cm with the solid vascular solid component measuring 2.3 × 2.4 cm. A single vascular supply to this solid component was confirmed with colour Doppler. By 30 + 2 weeks, the tumour measured 9 × 9 × 10 cm and was significantly larger than the fetal head (Fig. 3). A second MRI confirmed this incremental increase in size with associated downward displacement of the left ear and left orbit. There was significant scalloping of the calvarium and compression of the optic nerve (Fig. 4).

The case was discussed at the MDT which included input from paediatric radiology, paediatric otorhinolaryngology, fetal medicine, neonatology and paediatric neurosurgery. It was concluded that interval drainage of the cystic lesion may be beneficial for three reasons: firstly, to decrease the compressive effects on the developing orbit and optic nerve; secondly, to aid in diagnosis; and, lastly, to facilitate caesarean delivery of the fetal head. MDT discussion also included the timing of delivery and which members of the team should be present at delivery.

**Fig. 5:**
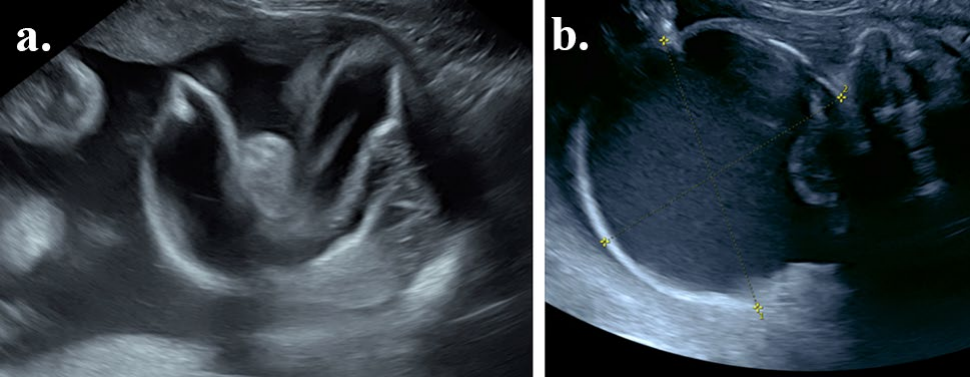
Ultrasound images of the lesion before and after drainage. **a** highlights the collapse of the cystic structure after the removal of 640 ml of light yellow coloured at 33 + 3 weeks gestation. **b** shows the reaccumulation by 34 + 3 weeks gestation

At 33 + 3 weeks, an uncomplicated drainage of the fetal tumour was performed using a 22 gauge needle and under ultrasound guidance. In total, 640 ml of clear fluid was drained. A good clinical result was obtained with the collapse of the cystic structure (Fig. 5a); however, almost immediately following the procedure, the mass began to reform. An ultrasound performed on day 1 post drainage revealed the lesion was already 8.2 × 8.5 × 5.5 cm and, within a week, had increased to 9 × 9 cm (Fig. 5b), almost its pre-drainage size. Cytopathological examination of the fluid revealed scanty lymphocytes and mononuclear cells only. The findings were non-diagnostic. As a result, it was deemed that further antenatal drainage would be of little benefit.

**Fig. 6:**
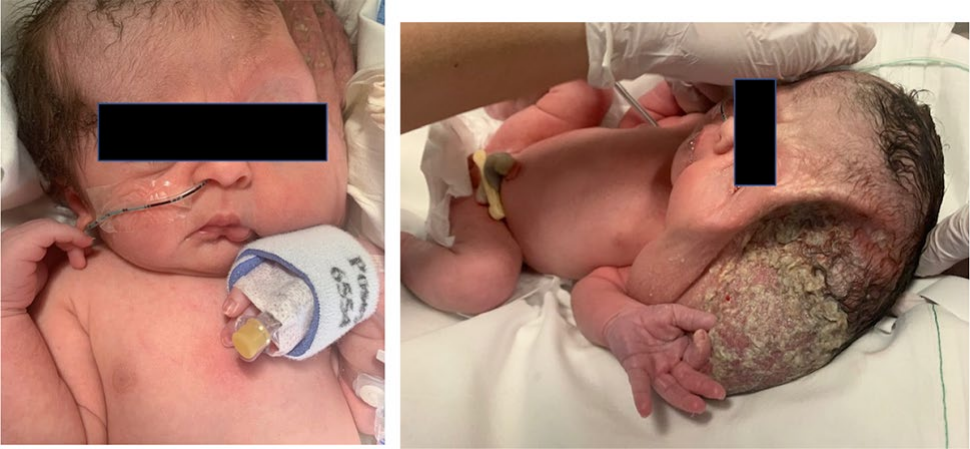
Images of the baby in the immediate postnatal period. This shows the large cystic lesion immediately after caesarean delivery. The cyst re-accumulated after drainage at the time of caesarean delivery

**Fig. 7:**
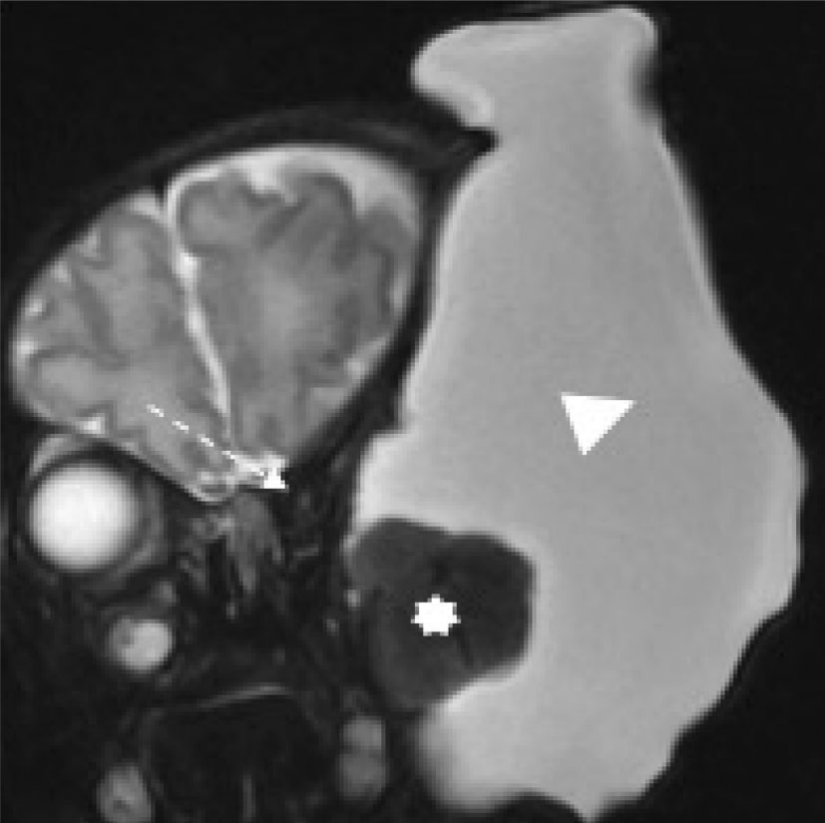
Postnatal MRI of the head and neck. Coronal T2-weighted MRI of the head and neck on day 1 of life again demonstrates the large left-sided cystic mass (arrowhead) with a solid component medially (star). There is distortion and displacement of adjacent structures including the left orbit (dashed arrow). Some decrease in the size of the cystic component is noted due to drainage a few hours previously postnatally

The MDT decided it best to drain the cyst peri-operatively to allow for safe caesarean delivery. At 38 + 1 weeks, an elective caesarean delivery was performed. Immediately prior to the uterine incision, 960 ml of cystic fluid was drained from the tumour under ultrasound guidance. A live female infant was delivered through a low transverse uterine incision with a birth weight of 3.12 kg (Fig. 6). The baby was dried and stimulated and was vigorous at birth with good respiratory effort. Its APGARS were 9 at 1 min and 9 at 5 min. Following delivery, serous fluid was draining from the puncture site, and the infant was commenced on IV gentamicin and benzylpenicillin. There was no lateral eye movement of the left eye. It is likely that the significant distortion of the orbit compromised the function of the intraorbital muscles and the abducens nerve. The left zygomatic arch was displaced postero-inferiorly, and the palate was intact, but it was high-arched. Almost immediately after birth, the cyst had refilled (Fig. 7), and the baby was transferred for further surgical management.

## Neonatal care

**Fig. 8:**
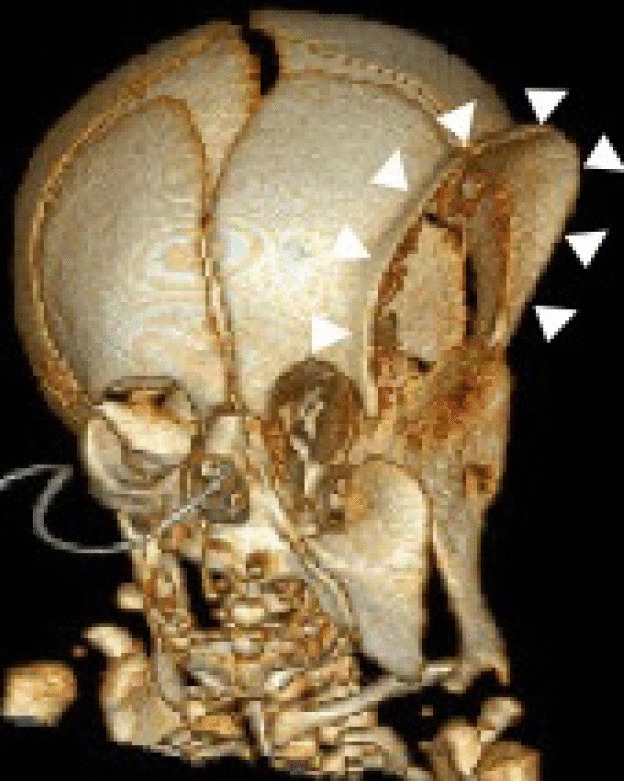
CT images of the head and neck. Bony 3D reformat of a CT of the head and neck demonstrates the extent of bony remodelling in the left frontal, parietal and temporal bones with extensive bony thinning and a prominent bony ridge (arrowheads) at the margins of the mass

Postnatally, the infant underwent a MRI (Fig. 7) which confirmed there was no intracranial connection between the cystic mass and the left temporal fossa. A decision was made to perform a simple resection of the cyst and contents in the infratemporal fossa to remove the pressure effects, determine the histological nature of the tissue and allow natural remodelling of the significant bony deformity. The extent of the bony deformity was assessed on postnatal CT imaging (Fig. 8). On day 29, an excision was performed of this large cystic mass having undergone two previous decompressions of the cyst by aspiration a few days beforehand. During the surgery, it was confirmed that there was no intracranial connection, and the assumption made was that the cystic fluid emanated from the tissue in the infratemporal fossa. Histology was reported as fragments of skeletal muscle and mature glial tissue with dystrophic calcification consistent with the diagnosis of a mature teratoma. There was no recollection post-operatively, and the infant made an uneventful recovery and was discharged 5 days later. A good postnatal outcome is expected.

## Discussion

This case highlights the significant challenges faced when a patient presents with a rare and complex case of a congenital giant fetal facial teratoma. A congenital tumour is one that is present at birth or develops in the first 3 months of life [1]. The prevalence of congenital fetal tumours is thought to be in the region of 1.7 to 13.5 per 100,000 live births [2]. The most common differential diagnosis when presented with an antenatal finding of a large mass in the fetal head and neck region includes teratomas, lymphatic malformations, branchial cyst/cleft remnant, scalp haemangioma and a lymphatic malformation [2–4]. Vascular lesions such as haemangiomas and lymphangiomas are relatively common whereas solid tumours such as teratomas are relatively uncommon [8]. A scalp haemangioma is a benign tumour of vascular endothelial cells [7] and is typically associated with increased colour flow on Doppler ultrasound [7]. Teratomas are benign congenital neoplasms which can have a combination of both cystic and solid components. A branchial remnant or branchial cyst is usually situated in the cervical region [5]. Rhabdomyomas can sometimes present in the fetal head or neck region [6], though these are rare and typically not cystic in nature.

Postnatal histopathological examination confirmed a cystic teratoma. Teratomas occur in approximately 0.74 per 10,000 live births [9]. Teratomas are usually present in the sacrococcygeal region and are generally benign congenital neoplasms with rare malignant transformation. Head and neck teratomas are said to occur 1 in 40,000 births [2, 10, 11] and account for about 5% of all teratomas [8], with only about 1.6% of all teratomas having a facial origin [8]. The teratoma is the most common neonatal solid tumour and is a germ cell tumour, originating from aberrant germ cells in the 4th or 5th week of gestation [12]. These germ cells proliferate and can become a mature or immature teratoma depending on the amount of neuroepithelium present (immature tissue) [12]. Teratomas usually contain some mature components of all 3 germ layers and also some immature neuroglial elements [2]. Intralesional fat is nearly pathognomonic of a teratoma, but this can be hard to discern in prenatal imaging including on MRI [2]. It usually presents as a hypoechoic cystic mass with an echogenic nodule known as the Rokitansky nodule which usually represents fat [16].

A teratoma’s heterogeneity and complex composition usually reflects its mature nature yet benign histology. Of note, immature tissue in a teratoma does not necessarily indicate malignancy as was previously assumed [2]. Benign mature teratomas tend to be cystic, whereas immature teratomas tend to be more solid in nature [2]. Teratomas are frequently highly vascular tumours and can sometimes be associated with haemodynamic instability [15].

Teratomas of the head and neck can lead to airway obstruction in the neonate [13]. Additional imaging in the form of antenatal MRI is a useful adjunct, not only to aid with diagnosis, but also to assess the potential risk of airway obstruction prior to delivery [14]. Recurrence of a benign resected teratoma is rare, and complete surgical resection of teratomas in the neonatal period improves longer-term survival [8, 15]. Karyotyping should be considered in cases of the fetal head and neck tumours as chromosomal anomalies can be present in up to 20% of cases [11]. Teratomas may be associated with trisomy 13, ring X-chromosome mosaicism, gonosomal pentasomy 49, gene mutations or abnormalities in early embryonic development.

The assessment of the fetal head and neck tumours involves a combination of ultrasound (US) and MRI imaging and genetic assessment as necessary. The use of ultrasound is still the modality of choice for imaging the fetus due to its speed, cost and availability. Due to the possibility for haemodynamic instability in teratomas [15], regular US assessment for evidence of hydrops is recommended as well as a detailed anatomical survey to exclude any other structural abnormalities. MRI is also useful to improve diagnostic accuracy and to assess other structures in more detail [17].

In this particular case, two MRI scans were performed to ensure a patent airway at delivery. The two most common types of congenital masses that obstruct the airway are the lymphatic malformation and the teratoma [18]. It was reassuring that there was no obvious intracranial extension or airway obstruction and that ex in utero intrapartum treatment (EXIT)/operation on placental support (OOPS) procedures were not necessary [11]. MRI has enhanced antenatal diagnosis of fetal abnormalities in recent years with the MERIDIAN cohort research study demonstrating that in 49% of cases, additional diagnostic information was provided by MRI, and additional prognostic information was yielded in 20% of cases [2]. In this case, one of the MRI reports suggested that there was compression of the optic nerve due to the mass effect on the lateral orbital and temporal regions of the left face. Comprehensive damage to the left optic nerve could result in unilateral blindness.

The survival rate of patients who presented with congenital fetal head and neck tumours is 35.35% [11]. EXIT procedures and surgical removal of the tumour are both associated with a good prognosis [11]. The current management of congenital head and neck masses includes sclerotherapy, surgery, medical therapy or a combination of them all (18).

This case highlights the challenges of diagnosing and managing rare and complex fetal facial tumours. The management of this particular case was complicated by the cystic mass persistently refilling almost immediately post drainage. Careful management and regular ultrasound scans were necessary to monitor the size of the mass and its compression effects on surrounding structures. MRI was a very useful modality to assess whether the EXIT procedure was necessary and also to aid in our understanding of the tumour’s boundaries and therefore guiding our care. Delivery of a child with a large facial tumour has been rarely reported in the literature and requires MDT management. A further challenge in this case is the paucity of data available on these rare fetal facial tumours. A good clinical outcome was obtained following multidisciplinary antenatal care and early referral to neurosurgical and ENT teams in a large tertiary paediatric hospital.

## Data Availability

Data available upon reasonable request from corresponding author.
